# A low-calorie balanced diet improves hepatic steatosis and preserves skeletal muscle in obesity: evidence from clinical and experimental studies

**DOI:** 10.1186/s13098-026-02242-9

**Published:** 2026-08-04

**Authors:** Ruijia Li, Zheng Yang, Yingyi Chen, Yanju Li, Haoyuan Xu, Zhiyong Rao

**Affiliations:** 1 West China Hospital, Sichuan University, Chengdu, 610041 China; 2 Data stored and provided by the Department of Clinical Nutrition and Gastroenterology, West China Hospital, Sichuan University, Sichuan, China

**Keywords:** Metabolic Dysfunction-Associated Steatotic Liver Disease, Low-calorie balanced diet, Obesity, Body composition analysis, skeletal muscle, hepatic steatosis

## Abstract

**Background:**

Metabolic dysfunction–associated steatotic liver disease (MASLD) is closely linked to obesity, insulin resistance, and endocrine dysregulation. Although calorie restriction is widely used for metabolic control, it is often accompanied by unintended skeletal muscle loss, which may negatively affect long-term metabolic outcomes. Therefore, identifying a dietary strategy that improves metabolic health while preserving muscle mass is of clinical importance.

**Methods:**

This study combined retrospective clinical observations with an in vivo high-fat diet (HFD)-induced obese mouse model to evaluate the effects of low-calorie balanced diet (LCD). Clinical data were used to assess changes in metabolic parameters and body composition. In parallel, obese mice subjected to LCD intervention were evaluated for body weight, glucose metabolism, hepatic steatosis, and skeletal muscle morphology. Gene expression related to hepatic lipid metabolism and muscle atrophy was analyzed.

**Results:**

LCD intervention improved metabolic parameters, reduced body weight gain, and alleviated hepatic lipid accumulation in both clinical observations and the animal model. In HFD-fed mice, LCD improved glucose homeostasis and partially reversed hepatic steatosis. Importantly, skeletal muscle structure was preserved, and the expression of muscle atrophy–related genes, including MSTN, FOXO3a, and MuRF1, was reduced. In the liver, LCD decreased the expression of lipogenic genes such as SREBP-1c, FASN, and ACC, while partially restoring fatty acid oxidation–related genes including PPARα and CPT1α.

**Conclusion:**

LCD improves metabolic dysfunction and hepatic steatosis while preserving skeletal muscle integrity in obesity. These findings provide translational evidence supporting LCD as a practical dietary strategy for MASLD management and highlight the importance of coordinated metabolic regulation between liver and skeletal muscle.

**Graphical abstract:**

**Figure:**
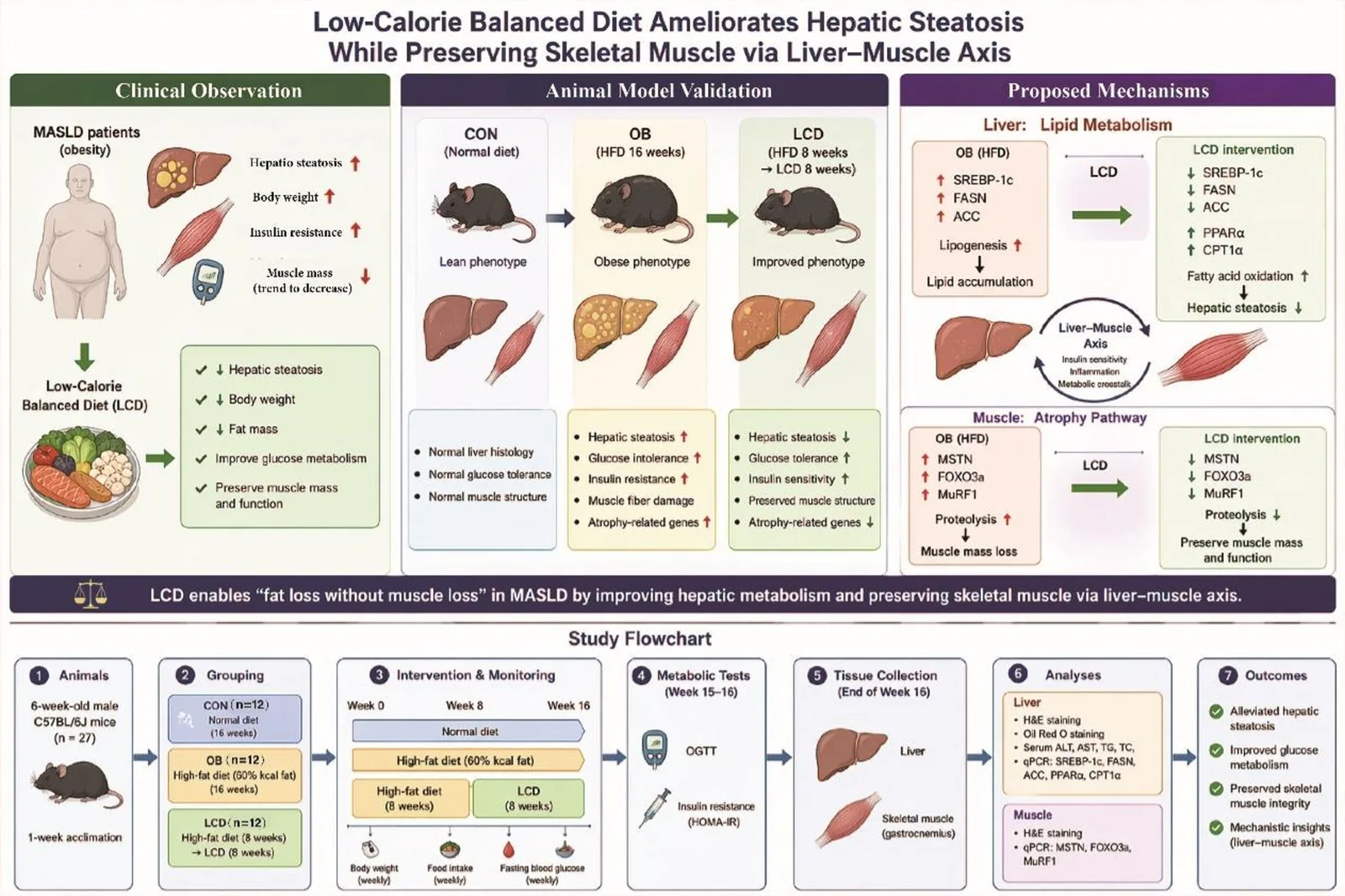

## Introduction

Metabolic dysfunction-associated steatotic liver disease (MASLD) has become the most prevalent chronic liver disorder worldwide, closely intertwined with obesity, insulin resistance and systemic metabolic disturbance [1]. Dietary intervention serves as the first-line non-pharmacological cornerstone for MASLD management, while conventional calorie-restricted weight-loss regimens frequently trigger unintended skeletal muscle catabolism alongside fat reduction [2]. Sarcopenia and skeletal muscle dysfunction further exacerbate insulin resistance, metabolic dysregulation, and hepatic metabolic deterioration, thereby worsening long-term clinical outcomes and compromising sustainable weight-loss efficacy in patients with obesity-associated MASLD. Moreover, reduced muscle strength has recently emerged as an independent predictor of poor clinical outcomes across several metabolic and hepatic disorders, further highlighting the importance of preserving skeletal muscle during dietary intervention [3, 4]. Thus, exploring a safe dietary strategy that simultaneously ameliorates hepatic steatosis, reduces adiposity and preserves skeletal muscle mass & function, remains an urgent clinical demand for MASLD precision nutritional management [5, 6].

Low-calorie balanced diet (LCD) features optimized calorie restriction matched with reasonable nutrient proportion, which has been widely applied in clinical weight management. Previous clinical evidence has confirmed that LCD effectively alleviates hepatic fat accumulation and metabolic abnormalities in MASLD patients. Several dietary strategies, including intermittent fasting, alternate-day fasting, time-restricted feeding, and Ramadan fasting, have demonstrated metabolic benefits in obesity and MASLD. However, these approaches mainly regulate meal timing rather than providing standardized nutritional composition, and their long-term adherence and effects on skeletal muscle preservation remain variable. In contrast, a low-calorie balanced diet provides controlled energy restriction while ensuring adequate intake of high-quality protein, essential fatty acids, vitamins, and minerals, thereby reducing the risk of excessive lean mass loss during weight reduction. Therefore, LCD represents a practical and clinically applicable dietary strategy for patients with obesity-associated MASLD. However, most existing studies only focus on hepatic and weight-loss outcomes, while longitudinal changes of skeletal muscle quantity, quality and function during LCD intervention [7], as well as the underlying mechanism linking dietary intervention, muscle homeostasis and hepatic metabolic improvement, remain poorly elucidated. In addition, clinical observational studies are limited by retrospective design, potential selection bias and confounding effects from lifestyle variables, which restricts robust causal inference of dietary efficacy.

Preclinical animal models can well recapitulate the pathological process of obesity-related MASLD, and provide reliable causal evidence and mechanistic explanation for clinical observational findings [8, 9]. Therefore, this study combined retrospective clinical cohort analysis and in vivo mouse experiment, to systematically investigate the effects of LCD on hepatic steatosis, body composition, glucose metabolism and skeletal muscle homeostasis in obese MASLD patients. Meanwhile, we further validated the phenotypic consistency of LCD intervention in high-fat diet induced obese fatty liver mice, and explored the potential molecular mechanism via skeletal muscle atrophy pathway and hepatic lipid metabolism gene expression [10]. We aimed to clarify the clinical value and preclinical basis of LCD in achieving fat reduction without muscle loss, and provide translational evidence for optimizing nutritional intervention guidelines for MASLD patients.

## Methods

### Clinical retrospective cohort study

#### Study subjects

A total of 119 obese patients diagnosed with MASLD who attended West China Hospital, Sichuan University from 2020 to 2024 were retrospectively enrolled. Patients were divided into LCD group (*n* = 94) and Non-LCD routine follow-up group (*n* = 25) according to whether they received standardized LCD nutritional guidance from the Department of Clinical Nutrition. Inclusion and exclusion criteria strictly conformed to MASLD diagnostic criteria and clinical nutritional intervention specifications. This study was approved by the Biomedical Ethics Review Committee of West China Hospital (Approval No. 2021 − 752). Written informed consent was waived due to retrospective anonymized clinical data analysis, in accordance with ethical regulations.

#### Intervention protocol

Patients in LCD group received personalized low-calorie balanced dietary intervention (800–1500 kcal/day, with optimal carbohydrate, protein and fat nutrient ratio), Participants were instructed to perform standardized exercise according to current WHO and American College of Sports Medicine recommendations, including at least 150 min/week of moderate-intensity aerobic exercise combined with resistance exercise involving major muscle groups at least two sessions per week. Patients in Non-LCD group maintained routine daily diet and identical exercise follow-up, without targeted calorie-restricted nutritional intervention. All subjects completed 9-month longitudinal follow-up, with clinical indicators collected at baseline, 3-month, 6-month and 9-month. The individualized LCD intervention was designed and supervised by registered clinical dietitians. Daily energy intake was reduced by approximately 500–750 kcal relative to estimated energy requirements. The dietary macronutrient distribution consisted of approximately 50–55% carbohydrate, 20–25% protein, and 25–30% fat, while ensuring adequate intake of dietary fiber, vitamins, and minerals. Participants received individualized dietary counseling throughout the intervention period. Compliance with the dietary prescription was assessed through regular outpatient follow-up and dietary records.

#### Outcome detection

Body composition indices including BMI, percentage body fat (PBF), body fat mass (BFM), visceral fat area (VFA), waist-to-hip ratio (WHR), skeletal muscle index (SMI) and skeletal muscle mass (SMM) were detected by bioelectrical impedance. Hepatic controlled attenuation parameter (CAP) was measured by transient elastography to quantify hepatic steatosis severity. Serum liver function indicators (ALT, AST) and blood lipid parameters (TG) were detected by routine clinical biochemical assay.

#### Statistical analysis

Statistical analyses were performed using SPSS version 22.0 (IBM Corp., Armonk, NY, USA). Continuous variables were first assessed for normality using the Shapiro–Wilk test. Normally distributed data with homogeneity of variance were analyzed using the independent-samples *t*-test, whereas Welch’s *t*-test was applied when the assumption of equal variances was violated. Non-normally distributed variables were compared using the Mann–Whitney *U* test.

For longitudinal clinical outcomes measured repeatedly during follow-up (e.g., monthly changes in visceral fat area), linear mixed-effects models were used to account for within-subject correlations over time. Baseline measurements, age, and sex were included as covariates to minimize the potential influence of baseline differences between groups. In addition, subgroup sensitivity analyses stratified by age, sex, and baseline body mass index (BMI) were performed to evaluate the robustness of the primary findings despite the unequal sample sizes between groups.All statistical tests were two-sided, and a *P* value < 0.05 was considered statistically significant.

## Inclusion and exclusion criteria for patients with MASLD

**Table:** 

Criteria	Details
**Inclusion**	- Confirmed steatotic liver disease (via histology, imaging, or blood biomarkers). - At least one metabolic risk factor: **i**. Overweight/obesity (BMI ≥ 23 kg/m² for Asians); **ii**. Type 2 diabetes mellitus (T2DM); **iii**. Metabolic dysfunction (≥ 2 metabolic risk abnormalities);
**Exclusion**	- Severe comorbidities (e.g., malignancy, tuberculosis); - Receiving total parenteral nutrition; - Liver disease due to viral hepatitis, drug-induced liver injury, or autoimmune liver diseases.; - Excessive alcohol intake: >140 g/week (males) or > 70 g/week (females) [11]; - Pregnancy, ascites, or implanted cardiac devices;

### Animal experimental study

#### Animals and diets

Male C57BL/6J mice (6 weeks old) were purchased from Beijing Vital River Laboratory Animal Technology Co., Ltd. (Beijing, China). All mice were housed in a specific pathogen–free (SPF) animal facility under controlled conditions (temperature 21–25 °C, humidity 40–70%, 12 h light/dark cycle). Mice were housed at four animals per cage under standard laboratory conditions with free access to water and the assigned experimental diets. After 1 week of adaptive feeding, mice were randomly divided into three groups (*n* = 12 per group): Control group (CON): fed standard chow diet for 16 consecutive weeks. Obese model group (OB): fed 60% high-fat diet (HFD) for 16 consecutive weeks to induce obesity and MASLD. Low-calorie balanced diet group (LCD): fed 60% HFD for 8 weeks to establish obesity and hepatic steatosis, then switched to low-calorie balanced diet for another 8 weeks.

Diet compositions (Shuyu Biotechnology, China): CON diet: total energy 3.66 kcal/g; 12.79% fat, 20.54% protein, 66.67% carbohydrates. OB diet: 60% fat-derived calories (high-fat diet). LCD diet: 10% fat, 30% protein, 60% carbohydrates.

Body weight was monitored weekly. Food intake was measured weekly per cage and normalized to the number of mice in each cage to calculate average daily food intake per mouse (g/mouse/day).

At the end of the 16-week experiment, mice were fasted overnight, anesthetized, and blood, liver, tibialis anterior (TA), soleus (SOL), gastrocnemius, and quadriceps muscle tissues were collected and stored at − 80 °C for subsequent analysis.

All animal procedures were approved by the Institutional Animal Care and Use Committee (IACUC) and the Animal Ethics Committee of West China Hospital, Sichuan University (Approval No. 20250218003), and were performed in accordance with institutional and national guidelines for the care and use of laboratory animals. At the end of the experimental period, mice were euthanized by gradual-fill carbon dioxide (CO₂) inhalation followed by cervical dislocation as a secondary physical method to ensure death. Animals were confirmed to be unconscious by the absence of spontaneous movement and pedal withdrawal reflex before tissue collection. All efforts were made to minimize animal suffering.

#### Glucose tolerance test (GTT)

After 12 h fasting, mice were intraperitoneally injected with glucose (1.0 g/kg body weight). Blood glucose was measured at 0, 15, 30, 60, 90, and 120 min using a glucometer. The area under the curve (AUC) was calculated for each mouse. Blood glucose concentrations were measured using an Accu-Chek Performa glucometer (Roche Diagnostics, Mannheim, Germany).

#### Insulin tolerance test (ITT)

After 4 h fasting, mice were intraperitoneally injected with insulin (0.75 U/kg body weight). Blood glucose was measured at 0, 15, 30, 60, 90, and 120 min. The AUC was calculated accordingly. Blood glucose concentrations were measured using an Accu-Chek Performa glucometer (Roche Diagnostics, Mannheim, Germany).

#### In Vivo Micro‑CT body composition analysis

Whole‑body micro‑CT scanning was performed to quantify fat mass, lean mass, skeletal muscle mass, and visceral fat volume. Images were analyzed using standard software to calculate total fat percentage, lean mass percentage, and skeletal muscle mass. Body composition was assessed using an in vivo micro-computed tomography system (Bruker Skyscan 1270, Germany). Mice were lightly anesthetized with isoflurane during image acquisition to minimize motion artifacts. Measurements were performed before dietary intervention and at the end of the experimental period.

#### Grip strength test

Forelimb grip strength was measured using a digital grip strength meter. Each mouse was tested three times consecutively; the maximum force was recorded and normalized to body weight.

#### Rotarod exercise endurance test

Muscle endurance was evaluated using an accelerating rotarod apparatus. The latency to fall was recorded as endurance time.

#### Blood biochemical analysis

Serum alanine transaminase (ALT), aspartate aminotransferase (AST), triglyceride (TG), total cholesterol (TC), high‑density lipoprotein cholesterol (HDL‑C), and low‑density lipoprotein cholesterol (LDL‑C) were measured using an automatic biochemical analyzer (Cobas 6000 c501, Roche Diagnostics, Switzerland). Serum free fatty acid (FFA) concentration was determined using a commercial mouse FFA ELISA kit (Nanjing Jiancheng Bioengineering Institute, China) following the manufacturer’s instructions.

#### Histopathological analysis

Liver and skeletal muscle tissues were fixed in 4% paraformaldehyde, embedded in paraffin, and sectioned at 5 μm. Hematoxylin and eosin (H&E) staining was performed to observe hepatic steatosis, inflammatory infiltration, and skeletal muscle fiber morphology. Hepatic steatosis was evaluated using both H&E staining and Oil Red O staining. Histological assessment was performed based on hepatocellular lipid vacuolation and lipid droplet area.

#### Real‑time quantitative PCR (qRT‑PCR)

Total RNA was extracted from liver and skeletal muscle tissues using TRIzol reagent (Thermo Fisher Scientific, USA). RNA concentration and purity were determined using a NanoDrop spectrophotometer. Equal amounts of RNA were reverse‑transcribed into cDNA using a HiScript II Q RT SuperMix kit (Vazyme, China). qRT‑PCR was performed on a Bio‑Rad CFX96 system using SYBR Green Master Mix (Vazyme, China). The relative gene expression was calculated using the 2 − ΔΔCt method with Rps18 as the internal reference. The primer sequences used in this study are provided in table.

**Table:** 

Gene	Sequences5’-3’	Species
*Rps18*	Forward: TTCGCCATCACTGCCATTAAGGG Reverse: ATCACTCGCTCCACCTCATCCTC	mouse
*Mstn*	Forward: GCACCAAGCAAACCCCAGAGG Reverse: AGCACCCACAGCGGTCTACTAC	mouse
*Mafbx*	Forward: CCAGACCTGCATGTGCTCAGTG Reverse: AACGCTTGCGAATCTGCCTCTC	mouse
*Murf-1*	Forward: AGTGTCCATGTCTGGAGGTCGTTT Reverse: ACTGGAGCACTCCTGCTTGTAGAT	mouse
*FOXO3a*	Forward: GGCAAGCACAGAGTTGGATG Reverse: CTGCTTTGCATTCAGTCCACT	mouse
*PPARα*	Forward: AGAGCCCCATCTGTCCTCTC Reverse: ACTGGTAGTCTGCAAAACCAAA	mouse
*SREBP-1c*	Forward: GGAGCCATGGATTGCACATT Reverse: GGCCCGGGAAGTCACTGT	mouse
*CPT1α*	Forward: CTCCGCCTGAGCCATGAAG Reverse: CACCAGTGATGATGCCATTCT	mouse
*ACC*	Forward: ATGGGCGGAATGGTCTCTTTC Reverse: TGGGGACCTTGTCTTCATCAT	mouse
*FASN*	Forward: GGAGGTGGTGATAGCCGGTAT Reverse: TGGGTAATCCATAGAGCCCAG	mouse

#### MQI (Muscle quality index)

MQI was defined as the ratio of skeletal muscle mass to total body weight.

#### Statistical analysis

All data are expressed as mean ± standard deviation (SD). One‑way ANOVA followed by Tukey’s post hoc test was used for multiple group comparisons. A value of *P* < 0.05 was considered statistically significant.

## Results

### Clinical outcomes of LCD intervention in MASLD patients

**Fig. 1:**
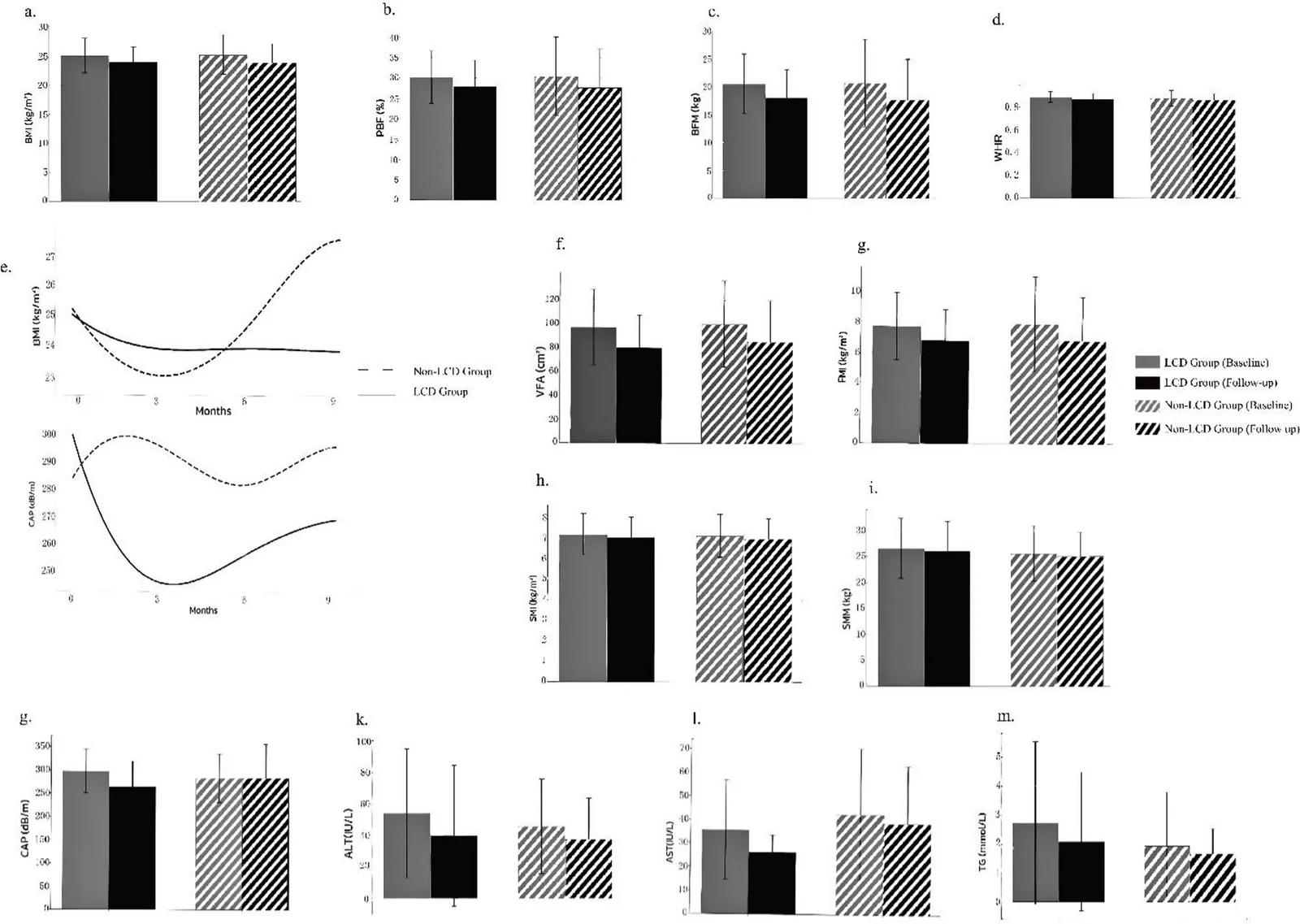
Effects of a Low-Calorie Diet Intervention on Body Composition and Metabolic Parameters in Overweight Individuals. (**a–d**) No significant differences were observed at baseline between the LCD and non-LCD groups in body mass index (BMI, kg/m²), percentage body fat (PBF, %), body fat mass (BFM, kg), and waist-to-hip ratio (WHR). (**e**) Longitudinal changes in BMI and controlled attenuation parameter (CAP, dB/m). The LCD group showed a rapid and sustained decrease, whereas the non-LCD group exhibited an initial decrease followed by rebound. (**f–g**) Visceral fat area (VFA, cm²) and fat mass index (FMI, kg/m²) were significantly reduced after LCD intervention, while minimal changes were observed in the non-LCD group. (**h–i**) Skeletal muscle index (SMI, kg/m²) and skeletal muscle mass (SMM, kg) showed no significant differences, suggesting that LCD did not adversely affect muscle mass. (**j**) Hepatic steatosis assessed by CAP (dB/m) was significantly improved in the LCD group. (**k–l**) Liver function markers alanine aminotransferase (ALT, U/L) and aspartate aminotransferase (AST, U/L) showed decreasing trends following LCD intervention. (**m**) Triglyceride (TG, mmol/L) levels were reduced more prominently in the LCD group.Data are presented as mean ± standard deviation (SD). Between-group comparisons were performed using independent t-tests or one-way ANOVA, and within-group comparisons were analyzed using paired t-tests. ns, not significant; *P < 0.05, **P < 0.01, ***P < 0.001, ****P < 0.0001

Baseline demographic, body composition, hepatic function and serum metabolic indicators were well balanced between LCD group and Non-LCD group (all *P* > 0.05) (Table 1). After 9-month longitudinal follow-up, LCD intervention significantly reduced body weight, BMI, visceral fat area, total body fat percentage, body fat mass and waist-to-hip ratio in MASLD patients. Notably, skeletal muscle mass and skeletal muscle index remained stable without significant decline throughout the whole intervention period, confirming the distinct muscle-sparing advantage of low-calorie balanced diet during fat loss. Meanwhile, CAP, serum ALT and AST levels were markedly decreased in LCD cohort, accompanied by remarkable improvement in serum triglyceride metabolism, indicating alleviated hepatic steatosis and liver injury (Table 2). Longitudinal dynamic analysis further revealed that the metabolic and hepatic protective effects of LCD emerged at 3-month follow-up, and maintained stable and durable efficacy during the entire 9-month observation (Table 3). In contrast, patients in Non-LCD group presented persistent deterioration of visceral adiposity, hepatic fat deposition and liver enzyme levels, without favorable metabolic improvements during follow-up (Fig.  1).

**Table 1:** Baseline Characteristics and Comparisons Between LCD and Non-LCD Groups in Patients with MASLD

variable	LCD group (n=94)	Non-LCD group(n=25)	P	Effect Size	95% CI
Age (years)	46.17±13.32	45.89±12.98	0.892	0.021	[-0.42, 0.46]
Sex (male/female)	71/23	18/7	0.765	φ=0.045	[-0.18, 0.27]
BMI (kg/m²)	25.14±2.98	25.33±3.40	0.802	0.059	[-0.38, 0.49]
CAP (dB/m)	298.15±47.45	283.36±51.93	0.181	0.301	[-0.11, 0.71]
Height (cm)	164.78±9.78	163.26±7.84	0.756	0.06	[-2.24, 3.44]
Weight (kg)	68.54±11.65	67.54±10.86	0.582	-0.10	[-0.34, 0.14]
SMM (kg)	26.53±5.78	25.56±5.35	0.673	-0.06	[-0.30, 0.18]
SMI (kg/m²)	7.24±1.01	7.20±1.05	0.625	-0.08	[-0.32, 0.16]
PBF (%)	30.22±6.43	30.51±9.66	0.471	-0.13	[-0.37, 0.11]
WHR	0.89±0.05	0.88±0.07	0.538	-0.11	[-0.35, 0.13]
VFA (cm²)	97.16±31.60	100.25±36.08	0.482	-0.12	[-0.36, 0.12]
TBI (μmol/L)	15.10±7.19	21.46±16.38	0.424	-0.14	[-0.38, 0.10]
DBIL (μmol/L)	3.95±2.62	8.82±12.92	0.513	-0.12	[-0.36, 0.12]
IBIL (μmol/L)	11.84±5.61	12.63±6.99	0.471	-0.13	[-0.37, 0.11]
ALT (U/L)	53.99±41.06	45.84±30.12	0.433	-0.14	[-0.38, 0.10]
ALP (U/L)	87.46±28.34	118.04±78.16	0.382	-0.15	[-0.39, 0.09]
AST (U/L)	35.62±21.11	42.56±27.75	0.521	-0.11	[-0.35, 0.13]
TP (g/L)	72.21±12.55	73.98±6.10	0.563	-0.10	[-0.34, 0.14]
ALB (g/L)	47.96±6.09	46.89±10.88	0.602	-0.09	[-0.33, 0.15]
CHOL (mmol/L)	4.95±1.23	4.72±1.70	0.478	-0.13	[-0.37, 0.11]
TG (mmol/L)	2.73±2.80	1.93±1.85	0.416	-0.14	[-0.38, 0.10]
HDL-C (mmol/L)	1.24±0.45	1.28±0.34	0.696	0.05	[-0.19, 0.29]
BFM (kg)	20.64±5.42	20.76±7.86	0.439	0.18	[-3.94, 1.34]
FMI (kg/m²)	7.69±2.21	7.91±3.14	0.518	0.15	[-1.32, 0.52]
LDL-C (mmol/L)	2.87±0.82	2.67±1.26	0.379	0.20	[-0.65, 0.25]

MASLD = Metabolic Dysfunction-Associated Steatotic Liver Disease; LCD = low-calorie balanced diet; BMI = body mass index; CAP = controlled attenuation parameter; SMM = skeletal muscle mass; SMI = skeletal muscle index; PBF = percentage of body fat; WHR = waist-to-hip ratio; VFA = visceral fat area; TBIL = total bilirubin; DBIL = direct bilirubin; IBIL = indirect bilirubin; ALT = alanine transaminase; ALP = alkaline phosphatase; AST = aspartate transaminase; TP = total protein; ALB = albumin; CHOL = total cholesterol; TG = triglycerides; HDL-C = high-density lipoprotein cholesterol; BFM = body fat mass; FMI = fat mass index; LDL-C = low-density lipoprotein cholesterol

### LCD Ameliorates systemic insulin resistance and metabolic disturbance in obese MASLD Mice

**Fig. 2:**
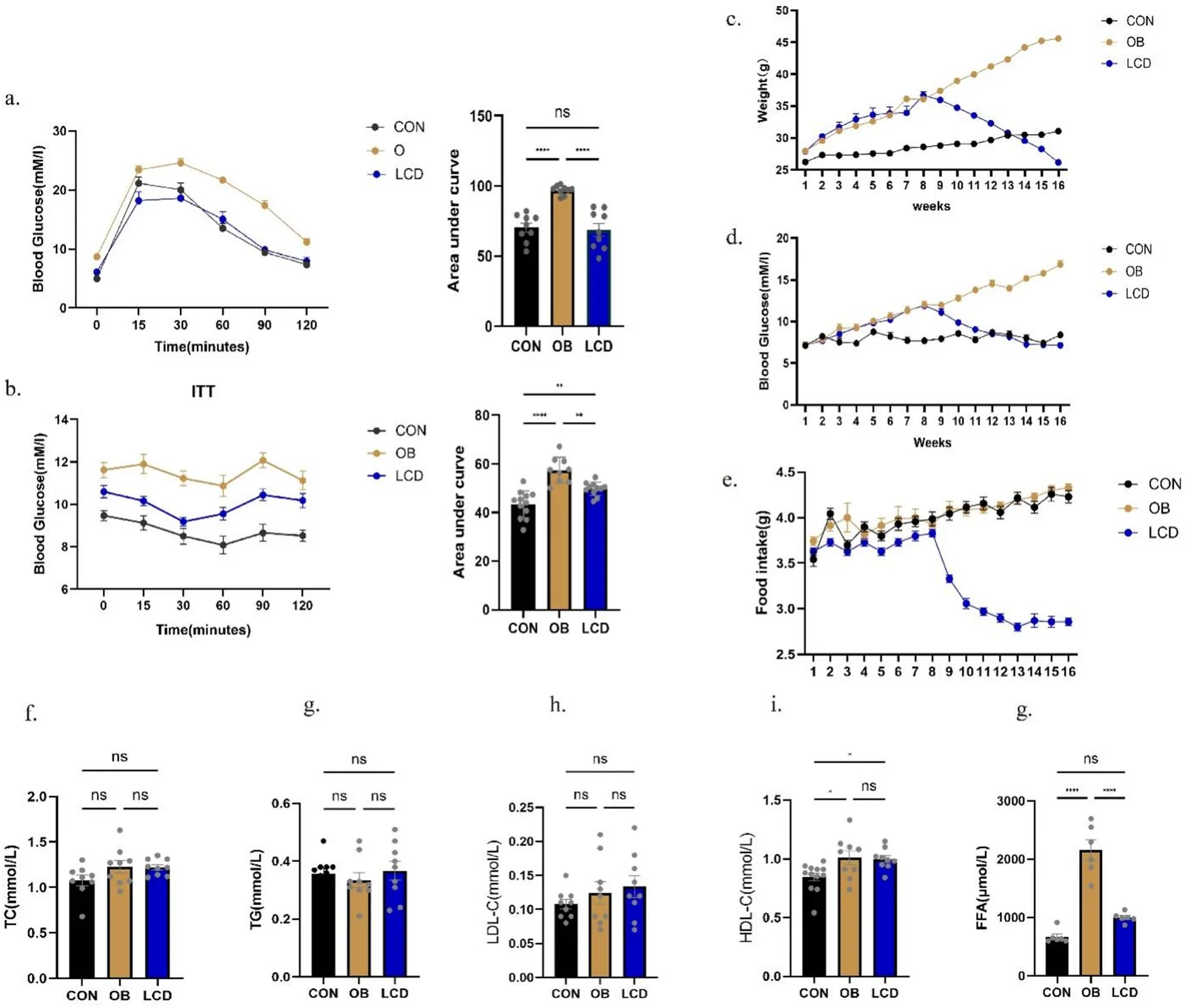
Low-calorie diet (LCD) ameliorates high-fat diet–induced metabolic dysfunction in mice. (**a**) Glucose tolerance test (GTT). The OB group exhibited significantly elevated blood glucose levels and delayed clearance, while LCD intervention markedly improved glucose tolerance. Area under the curve (AUC) quantification is shown on the right. (**b**) Insulin tolerance test (ITT). The OB group displayed insulin resistance, which was partially restored by LCD intervention. AUC analysis is presented on the right. (**c**) Body weight changes over 16 weeks. The OB group showed continuous weight gain, whereas the LCD group exhibited progressive weight loss after dietary intervention starting at week 8. The CON group remained stable. (**d**) Fasting blood glucose levels over time. Blood glucose progressively increased in the OB group, while LCD intervention significantly reduced glucose levels. (**e**) Food intake (g/mouse/day). A marked reduction in food intake was observed in the LCD group after week 8, whereas CON and OB groups showed a slight increase over time. (**f–h**) Serum lipid profiles, including total cholesterol (TC), triglycerides (TG), and low-density lipoprotein cholesterol (LDL-C), showed no significant differences among groups. (**i**) High-density lipoprotein cholesterol (HDL-C) showed a mild increase in the OB group and partial normalization after LCD intervention. (**j**) Free fatty acids (FFA) were significantly elevated in the OB group and markedly reduced following LCD intervention. Data are presented as mean ± standard deviation (SD), with n = 6–8 mice per group. Statistical analysis was performed using one-way ANOVA followed by Tukey’s post hoc test. Time-course data were analyzed using two-way repeated-measures ANOVA. ns, not significant; *P < 0.05, **P < 0.01, ***P < 0.001, ****P < 0.0001

As shown in Fig. 2, long-term high-fat diet feeding induced obvious excessive weight gain and severe glucose metabolic disorder in OB model mice. GTT and ITT results demonstrated significantly impaired glucose tolerance and insulin sensitivity in OB group, with notably higher AUC values compared with normal CON mice. After 8-week LCD intervention, abnormal excessive body weight gain was prominently reversed, and systemic insulin resistance was markedly alleviated, with glucose tolerance and insulin response largely restored to near-normal physiological levels. For serum lipid profiles, OB mice exhibited abnormally elevated free fatty acid levels, while LCD treatment effectively corrected disordered systemic lipid metabolism and reduced elevated circulating free fatty acids and ectopic intracellular lipid deposition in liver and muscle tissues. No significant differences in TC, TG and LDL-C levels were detected among groups, and HDL-C levels were slightly improved following LCD intervention. Dynamic food intake monitoring confirmed that dietary intervention stably reduced caloric intake in LCD mice without abnormal starvation-induced metabolic stress.

### LCD preserves skeletal muscle mass, function and inhibits pathological muscle atrophy in vivo

**Fig. 3:**
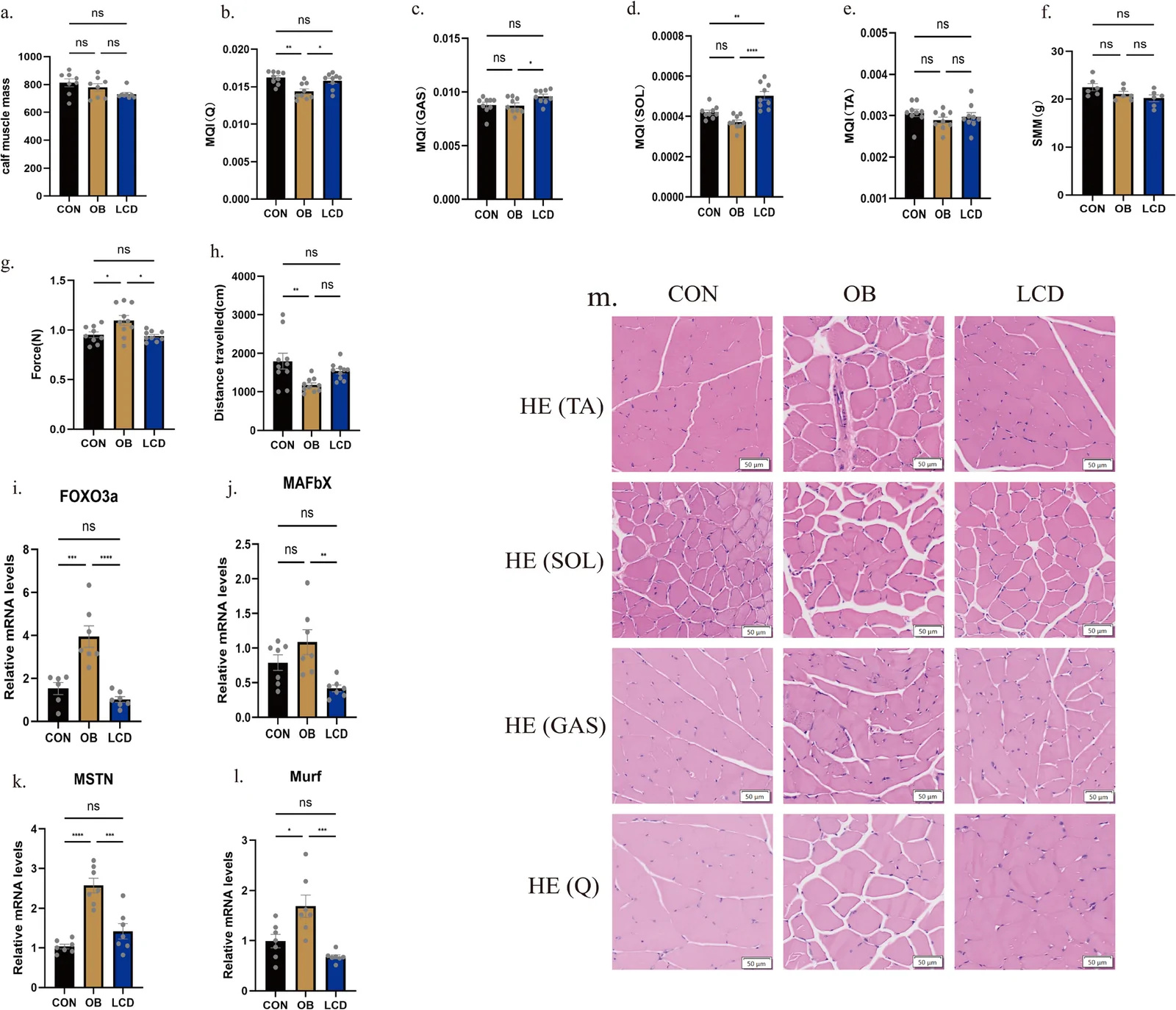
Low-calorie diet (LCD) improves skeletal muscle function and suppresses muscle atrophy–related signaling pathways. (**a**) Calf muscle mass showed no significant differences among groups. (**b–e)** Muscle quality index (MQI) of different muscles, including quadriceps (Q), gastrocnemius (GAS), soleus (SOL), and tibialis anterior (TA). The OB group exhibited reduced MQI in certain muscles, which was partially restored by LCD intervention. (**f**) Total skeletal muscle mass (SMM, **g**) showed no significant differences among groups. (**g**) Grip strength (Force, N) was reduced in the OB group and partially improved in the LCD group. (**h**) Spontaneous physical activity (distance traveled, cm) was significantly decreased in the OB group and improved following LCD intervention. (**i–l**) mRNA expression of muscle atrophy–related genes, including FOXO3a, MAFbx (Atrogin-1), MSTN, and MuRF1. These genes were significantly upregulated in the OB group and markedly downregulated after LCD intervention, indicating suppression of muscle catabolism. (**m**) Representative hematoxylin and eosin (H&E) staining of skeletal muscle (scale bar: 50 μm). The OB group showed disorganized muscle fibers and increased inter-fiber spacing, indicating structural damage, whereas LCD intervention improved muscle morphology. Muscles analyzed include tibialis anterior (TA), soleus (SOL), gastrocnemius (GAS), and quadriceps (**Q**). Data are presented as mean ± standard deviation (SD), with *n* = 6–8 mice per group. Statistical analysis was performed using one-way ANOVA followed by Tukey’s post hoc test. ns, not significant; *P < 0.05, **P < 0.01, ***P < 0.001

Micro-CT body composition analysis (Fig. 3) showed that obese MASLD mice presented declined muscle quality index and impaired skeletal muscle homeostasis. Functional tests further verified significantly weakened forelimb grip strength and shortened exhaustive exercise endurance in OB mice. H&E histological staining displayed markedly atrophied, narrowed skeletal muscle fiber morphology in TA, SOL, GAS and quadriceps muscle of obese model mice. Following LCD intervention, skeletal muscle quality, grip strength and exercise endurance function were significantly recovered, and normal muscle fiber morphology and structural integrity were maintained. qRT-PCR detection further revealed that key muscle atrophy genes including Foxo3a, MAFbx, Murf1 and muscle inhibitory factor Mstn were excessively upregulated in skeletal muscle of OB mice. LCD treatment notably suppressed abnormal activation of FOXO/UPS ubiquitin proteasome atrophy pathway, and downregulated mRNA expression of atrophy-related genes, thereby blocking pathological skeletal muscle degradation and achieving stable muscle mass preservation during calorie restriction (Fig. 3).

**Table 2:** Changes in body composition, hepatic, and metabolic parameters from baseline to follow-up $(\overline X\pm s)$

Variable	LCD group			non-LCD group			P*	P**
	Baseline	Follow-up	P	Baseline	Follow-up	P		
Height (cm)	164.78±9.78	164.72±9.83	0.971	163.26±7.84	163.26±7.84	1.00	0.479	0.496
Weight (kg)	68.54±11.65	65.34±11.12	0.057	67.54±10.86	63.71±9.90	0.19	0.551	0.309
BFM (kg)	20.64±5.42	18.16±5.02	0.001	20.76±7.86	17.79±7.30	0.182	0.946	0.308
SMM (kg)	26.53±5.78	26.05±5.67	0.573	25.56±5.35	24.93±4.78	0.669	0.493	0.407
BMI (kg/m²)	25.14±2.98	23.97±2.66	0.009	25.33±3.40	23.91±3.27	0.146	0.802	0.575
PBF (%)	30.22±6.43	27.90±6.48	0.023	30.51±9.66	27.63±9.47	0.302	0.495	0.610
FMI (kg/m²)	7.69±2.21	6.76±2.03	0.004	7.91±3.14	6.80±2.91	0.21	0.748	0.860
SMI (kg/m²)	7.24±1.01	7.10±0.99	0.351	7.20±1.05	7.03±0.99	0.521	0.717	0.691
WHR	0.89±0.05	0.87±0.05	0.003	0.88±0.07	0.86±0.06	0.259	0.561	0.763
VFA (cm²)	97.16±31.60	80.34±26.88	0.000	100.25±36.08	85.44±34.31	0.152	0.708	0.830
TBIL (mol/L)	15.10±7.19	17.21±12.76	0.967	21.46±16.38	22.85±20.51	0.954	0.168	0.310
DBIL (mol/L)	3.95±2.62	4.56±5.40	0.931	8.82±12.92	10.69±16.92	0.907	0.132	0.252
IBIL (mol/L)	11.84±5.61	11.83±6.75	0.477	12.63±6.99	12.16±5.41	0.892	0.52	0.518
ALT(IU/L)	53.99±41.06	39.71±44.80	0.001	45.84±30.12	37.36±26.38	0.327	0.436	0.727
AST(IU/L)	35.62±21.11	31.07±40.51	0.001	42.56±27.75	38.28±24.53	0.168	0.111	0.082
ALP(IU/L)	87.46±28.34	84.25±28.47	0.365	118.04±78.16	122.65±71.21	0.892	0.004	0.004
TP(g/L)	72.21±12.55	74.60±4.36	0.594	73.98±6.10	73.59±5.39	0.815	0.863	0.295
ALB(g/L)	47.96±6.09	48.25±3.22	0.944	46.89±10.88	44.13±5.91	0.497	0.038	0.002
CHOL (mmol/L)	4.95±1.23	4.76±0.98	0.147	4.72±1.70	4.49±1.25	0.599	0.395	0.249
TG (mmol/L)	2.73±2.80	2.09±2.39	0.009	1.93±1.85	1.64±0.89	0.641	0.028	0.732
HDL-C(mmol/L)	1.24±0.45	1.25±0.33	0.256	1.28±0.34	1.21±0.36	0.426	0.402	0.867
LDL-C(mmol/L)	2.87±0.82	2.77±0.83	0.420	2.67±1.26	2.61±1.05	0.849	0.463	0.357
CAP (dB/m)	298.15±47.45	251.44±48.42	0.000	283.36±51.93	285.55±73.73	0.906	0.181	0.032

* is a baseline comparison of the LCD and non-LCD groups, **is a comparison of the LCD group with the non-LCD group after the intervention.MASLD = Metabolic Dysfunction-Associated Steatotic Liver Disease; LCD = low-calorie balanced diet; BMI = body mass index; CAP = controlled attenuation parameter; SMM = skeletal muscle mass; SMI = skeletal muscle index; PBF = percentage of body fat; WHR = waist-to-hip ratio; VFA = visceral fat area; TBIL = total bilirubin; DBIL = direct bilirubin; IBIL = indirect bilirubin; ALT = alanine transaminase; ALP = alkaline phosphatase; AST = aspartate transaminase; TP = total protein; ALB = albumin; CHOL = total cholesterol; TG = triglycerides; HDL-C = high-density lipoprotein cholesterol; BFM = body fat mass; FMI = fat mass index; LDL-C = low-density lipoprotein cholesterol

### LCD Alleviates Hepatic Steatosis and Remodels Disordered Hepatic Lipid Metabolism

**Fig. 4:**
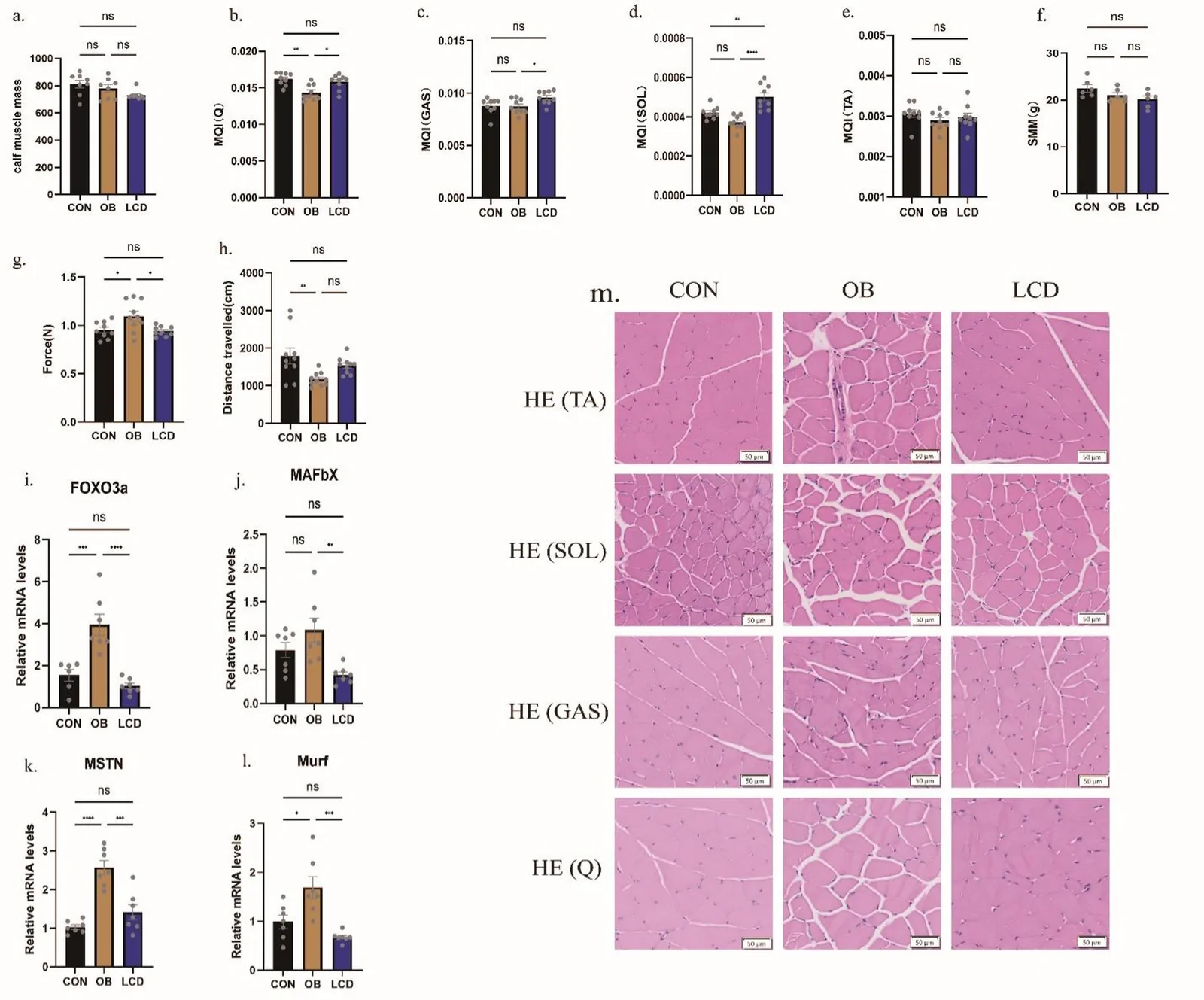
(**a–b**) Serum levels of alanine aminotransferase (ALT, U/L) and aspartate aminotransferase (AST, U/L) showed no significant differences among groups. (**c**) Liver index was increased in the OB group and showed a decreasing trend following LCD intervention. (**d**) Quantification of Oil Red O staining revealed a significant increase in lipid droplet area in the OB group, which was markedly reduced by LCD intervention. (**e**) Representative hematoxylin and eosin (H&E) and Oil Red O staining of liver tissue (scale bar: 50 μm). The OB group exhibited pronounced lipid vacuolation and accumulation, whereas LCD markedly alleviated hepatic steatosis. (**f**–**j**) Hepatic mRNA expression of lipid metabolism–related genes. Fatty acid oxidation genes (PPARα and CPT1α) were downregulated in the OB group and upregulated following LCD intervention. In contrast, lipogenesis-related genes (SREBP-1c, ACC, and FASN) were significantly upregulated in the OB group and suppressed after LCD treatment. Data are presented as mean ± standard deviation (SD), with n = 6–8 mice per group. Statistical analysis was performed using one-way ANOVA followed by Tukey’s post hoc test. ns, not significant; *P < 0.05, **P < 0.01, ***P < 0.001, ****P < 0.0001.

Serum liver function indicators ALT and AST were abnormally elevated in obese MASLD model mice. Liver H&E and Oil Red O staining revealed massive intracellular lipid droplet deposition and severe hepatic steatosis in OB group, accompanied by significantly increased liver index and quantitative hepatic lipid droplet area. After LCD nutritional intervention, pathological hepatic lipid accumulation was markedly reduced, hepatic histological damage was prominently alleviated, and liver morphological structure was restored. Mechanistic mRNA expression analysis showed that hepatic fatty acid oxidation genes (Pparα, Cpt1α) were suppressed, while de novo lipogenesis genes (Srebp-1c, Acc, Fasn) were abnormally activated in fatty liver mice. LCD intervention significantly upregulated fatty acid oxidative catabolism, inhibited excessive hepatic lipid synthesis, and reconstructed balanced hepatic lipid metabolic homeostasis, thus directly alleviating pathological progression of MASLD (Fig. 4).

**Table 3:** Longitudinal changes in selected parameters across 9-month follow-up$(\overline X\pm s)$

Variable	Groups	Baseline	3 monthsfollow-up	P	6 monthsfollow-up	P	9 monthsfollow-up	P
TBIL(mol/L)	LG	15.10±7.19	15.61±10.74	0.516	20.37±15.35	0.092	12.29±5.08	0.071
	NLG	21.46±16.38	16.99±11.11	0.476	22.65±13.62	0.523	15.60±3.55	0.746
DBIL(mol/L)	LG	3.95±2.62	4.56±6.58	0.350	4.59±2.98	0.207	2.95±1.14	0.057
	NLG	8.82±12.92	5.83±6.52	0.443	9.82±10.86	0.443	4.24±1.40	0.911
IBIL(mol/L)	LG	11.84±5.61	11.06±5.57	0.344	13.36±7.78	0.489	9.33±4.16	0.033
	NLG	12.63±6.99	11.16±5.21	0.401	12.83±3.93	0.742	11.36±2.31	0.867
ALT(IU/L)	LG	53.99±41.06	44.13±54.23	0.005	39.59±25.85	0.074	36.45±19.07	0.107
	NLG	45.84±30.12	29.80±19.82	0.149	39.90±25.05	0.534	59.00±36.73	0.330
AST(IU/L)	LG	35.62±21.11	34.27±49.13	0.010	28.66±9.99	0.131	25.85±6.01	0.049
	NLG	42.56±27.75	35.30±20.42	0.249	41.40±23.65	0.956	32.60±9.41	0.373
ALP(IU/L)	LG	85.42±104.50	57.12±59.52	0.032	77.78±100.64	0.352	41.45±38.25	0.030
	NLG	95.00±124.58	94.60±107.46	0.622	65.00±59.29	0.315	103.60±86.09	0.559
TP(g/L)	LG	72.21±12.55	73.88±4.43	0.422	75.91±4.02	0.095	75.29±3.80	0.313
	NLG	73.98±6.10	75.14±5.16	0.610	71.94±9.52	0.470	76.30±2.55	0.426
ALB(g/L)	LG	47.96±6.09	48.20±3.16	0.794	48.42±4.25	0.420	48.85±2.65	0.445
	NLG	46.89±10.88	43.73±5.13	0.258	42.50±7.67	0.443	49.12±0.49	0.066
CHOL(mmol/L)	LG	4.95±1.23	4.76±0.98	0.185	4.80±0.98	0.373	5.06±0.91	0.658
	NLG	4.72±1.70	4.56±1.18	0.788	3.86±0.92	0.150	5.14±0.94	0.606
TG(mmol/L)	LG	2.73±2.80	2.03±2.50	0.006	1.89±1.71	0.101	2.26±1.87	0.412
	NLG	1.93±1.85	1.47±0.52	0.742	1.38±0.98	0.488	2.22±1.12	0.385
HDL-C(mmol/L)	LG	1.24±0.45	1.29±0.35	0.105	1.31±0.38	0.228	1.20±0.30	0.876
	NLG	1.28±0.34	1.20±0.19	0.701	1.28±0.33	0.913	1.09±0.16	0.210
LDL-C(mmol/L)	LG	2.87±0.82	2.75±0.77	0.373	2.81±0.80	0.704	3.04±1.03	0.436
	NLG	2.67±1.26	2.83±0.96	0.730	2.08±0.73	0.182	3.19±0.79	0.396
CAP(dB/m)	LG	298.15±47.45	248.20±43.89	0.000	257.20±36.59	0.000	269.24±56.13	0.020
	NLG	283.36±51.93	294.84±61.98	0.592	281.37±62.70	0.926	294.32±32.32	0.664
Weight(kg)	LG	68.54±11.65	64.29±9.96	0.020	65.51±11.36	0.206	65.29±8.96	0.246
	NLG	67.54±10.86	62.98±5.97	0.281	63.62±9.03	0.443	78.60±8.57	0.027
BFM(kg)	LG	20.64±5.42	17.68±4.88	0.000	18.90±5.32	0.120	18.50±4.18	0.101
	NLG	20.76±7.86	18.15±5.71	0.361	17.15±7.61	0.238	24.56±7.10	0.341
SMM(kg)	LG	26.53±5.78	25.73±5.24	0.382	25.70±6.16	0.477	25.85±4.90	0.605
	NLG	25.56±5.35	24.36±4.25	0.571	25.04±5.08	0.826	30.04±3.19	0.106
BMI(kg/m²)	LG	25.14±2.98	24.00±2.70	0.027	24.02±2.89	0.071	23.94±2.11	0.106
	NLG	25.33±3.40	23.11±2.15	0.072	24.67±3.10	0.608	27.60±2.86	0.188
PBF(%)	LG	30.22±6.43	27.61±6.43	0.016	29.11±7.35	0.468	28.43±6.03	0.377
	NLG	30.51±9.66	28.80±8.50	0.638	26.84±11.21	0.354	30.86±6.29	0.941
FMI(kg/m²)	LG	7.69±2.21	6.70±2.09	0.005	7.10±2.37	0.205	6.85±1.68	0.112
	NLG	7.91±3.14	6.80±2.42	0.335	6.87±3.29	0.401	8.64±2.48	0.640
SMI(kg/m²)	LG	7.24±1.01	7.10±0.96	0.405	7.00±1.01	0.255	7.06±0.86	0.400
	NLG	7.20±1.05	6.74±0.71	0.297	7.23±1.07	0.942	7.98±0.70	0.172
WHR	LG	0.89±0.05	0.86±0.04	0.000	0.87±0.04	0.044	0.87±0.05	0.175
	NLG	0.88±0.07	0.88±0.04	0.674	0.84±0.06	0.148	0.90±0.04	0.539
VFA(cm²)	LG	97.16±31.60	79.57±27.36	0.000	87.88±29.85	0.207	78.93±23.22	0.033
	NLG	100.25±36.08	89.10±29.53	0.405	90.91±38.86	0.516	113.08±39.70	0.496

MASLD = Metabolic Dysfunction-Associated Steatotic Liver Disease; LCD = low-calorie balanced diet; BMI = body mass index; CAP = controlled attenuation parameter; SMM = skeletal muscle mass; SMI = skeletal muscle index; PBF = percentage of body fat; WHR = waist-to-hip ratio; VFA = visceral fat area; TBIL = total bilirubin; DBIL = direct bilirubin; IBIL = indirect bilirubin; ALT = alanine transaminase; ALP = alkaline phosphatase; AST = aspartate transaminase; TP = total protein; ALB = albumin; CHOL = total cholesterol; TG = triglycerides; HDL-C = high-density lipoprotein cholesterol; BFM = body fat mass; FMI = fat mass index; LDL-C = low-density lipoprotein cholesterol

## Discussion

Clinical retrospective observations have indicated that structured dietary interventions, particularly calorie restriction with balanced macronutrient composition, can significantly improve metabolic dysfunction–associated steatotic liver disease (MASLD), while minimizing loss of skeletal muscle mass [12–14]. These clinical findings are supported by extensive evidence demonstrating that lifestyle modification remains the cornerstone therapy for MASLD [15, 16]. Notably, an important but often overlooked aspect of dietary intervention is the ability to simultaneously improve hepatic metabolism while preserving skeletal muscle mass [17, 18], which is critical for long-term metabolic health and prevention of sarcopenia-associated complications [19].

In the present study, a high-fat diet (HFD)-induced obese mouse model recapitulated key metabolic features, including obesity, hyperglycemia, insulin resistance, and hepatic lipid accumulation, which are widely recognized hallmarks of MASLD progression [20, 21]. Importantly, low-calorie diet (LCD) intervention partially reversed these metabolic abnormalities, reinforcing the translational relevance of dietary energy restriction observed in clinical settings [8, 22]. Beyond general metabolic improvement, our findings suggest that LCD exerts a dual-benefit effect by correcting hepatic lipid dysregulation while concurrently attenuating muscle catabolic signaling, highlighting its advantage over simple caloric restriction strategies that may induce muscle loss. Notably, unlike severe caloric restriction that may induce lean mass loss, the balanced LCD applied in this study appears to preserve skeletal muscle integrity while improving hepatic metabolism, highlighting the importance of macronutrient composition in mitigating the adverse effects of energy restriction. Notably, food intake was lower in the LCD group than in the OB group during the intervention period. This finding reflects not only reduced caloric intake but also the lower energy density and controlled formulation of the LCD. Therefore, the beneficial effects observed in the present study are likely attributable to the combined influence of caloric restriction and optimized nutrient composition rather than food quantity alone. Future studies using pair-feeding designs would help further distinguish the independent effects of dietary composition from reduced energy intake.

At the hepatic level, HFD feeding induced classical steatotic changes characterized by excessive lipid droplet accumulation and hepatocellular injury, consistent with established histopathological features of MASLD. Mechanistically, hepatic steatosis is driven by an imbalance between lipid acquisition and disposal, in which increased de novo lipogenesis and impaired fatty acid oxidation play central roles. In this context, transcription factors such as SREBP-1c and its downstream targets, including FASN and ACC [23], are key regulators of lipogenic pathways. Conversely, PPARα and CPT1α are essential mediators of mitochondrial fatty acid β-oxidation [24–26]. The observed upregulation of lipogenic genes together with suppression of oxidative pathways in HFD-fed mice reflects a metabolic shift favoring lipid accumulation, which is a core mechanism underlying MASLD. Notably, LCD intervention partially restored this transcriptional imbalance, suggesting that balanced caloric restriction not only reduces energy surplus but actively reprograms hepatic lipid metabolism toward a more oxidative phenotype, thereby improving liver function without inducing excessive metabolic stress.

Beyond hepatic metabolism, skeletal muscle alterations represent an important but often underappreciated component of metabolic dysfunction. Accumulating evidence indicates that obesity and insulin resistance are associated with impaired muscle structure and function, including increased lipid infiltration and reduced contractile capacity. In the present study, HFD-fed mice exhibited disrupted muscle architecture and increased expression of atrophy-related genes, including MSTN, FOXO3a, and MuRF1. These findings are consistent with previous studies demonstrating that activation of the FOXO signaling pathway promotes proteolysis through the ubiquitin–proteasome system. Myostatin, a negative regulator of muscle growth, has also been implicated in metabolic disease–associated muscle wasting [27–29].

Importantly, LCD intervention attenuated these molecular changes and improved muscle morphology, suggesting a protective effect against muscle catabolism. This finding is particularly significant, as conventional calorie restriction is often associated with loss of lean mass. In contrast, the present study demonstrates that a low-calorie diet with balanced macronutrient composition may preserve skeletal muscle by suppressing catabolic signaling pathways, potentially through improved insulin sensitivity and reduced systemic inflammation. This indicates that diet quality, rather than calorie reduction alone, is a critical determinant of muscle preservation during metabolic intervention.

Emerging evidence highlights the existence of a bidirectional liver–muscle axis that plays a critical role in systemic metabolic regulation [30, 31]. The liver and skeletal muscle communicate through circulating factors, including hepatokines and myokines, which modulate insulin sensitivity, inflammation, and energy metabolism. Within this framework, the present findings support a model in which LCD-induced improvements in hepatic metabolism reduce systemic lipotoxicity and inflammatory signaling, thereby alleviating muscle degradation, while preserved muscle mass further enhances glucose utilization and metabolic flexibility. This coordinated response underscores the importance of integrated organ crosstalk in mediating the therapeutic effects of dietary intervention.

Several limitations should be acknowledged. First, the clinical study was retrospective and conducted at a single center with an unequal sample size between the LCD (*n* = 94) and non-LCD (*n* = 25) groups, which may have reduced statistical power and introduced selection bias despite comparable baseline characteristics. Second, habitual physical activity and dietary adherence outside the prescribed intervention were not quantitatively assessed and therefore cannot be completely excluded as potential confounders. Third, mechanistic investigations were limited to gene expression analysis, and future studies should incorporate protein-level validation and functional experiments. Finally, prospective randomized clinical trials with larger sample sizes are warranted to further validate these findings. Additionally, subgroup sensitivity analyses were performed to verify the robustness of primary outcomes and mitigate potential selection bias caused by unequal sample sizes between the two clinical cohorts.

In conclusion, a low-calorie balanced diet improves metabolic dysfunction by reducing hepatic lipid accumulation, enhancing insulin sensitivity, and preserving skeletal muscle integrity. These effects are mediated through coordinated regulation of hepatic lipid metabolism, suppression of muscle atrophy pathways, and systemic metabolic crosstalk along the liver–muscle axis. Importantly, the present study highlights that effective dietary intervention should not only target hepatic steatosis but also maintain skeletal muscle mass, thereby providing a more comprehensive strategy for MASLD management. Future large-scale prospective studies are warranted to further validate these observations. In addition to preserving skeletal muscle mass, LCD also improved muscle strength. Improvements in muscle force may result from enhanced insulin sensitivity, reduced intramuscular lipid accumulation, improved mitochondrial function, and attenuation of muscle protein degradation. Collectively, these adaptations may improve muscle quality despite relatively modest changes in absolute muscle mass.

## Data Availability

The raw clinical data are not publicly available due to privacy and ethical restrictions but are available from the corresponding author upon reasonable request. All animal‑derived raw data generated in this study are available from the corresponding author on reasonable request.
